# Network analyses of internet gaming disorder symptoms and their links with different types of motivation

**DOI:** 10.1186/s12888-022-03708-6

**Published:** 2022-01-31

**Authors:** Rapson Gomez, Vasileios Stavropoulos, Deon Tullett-Prado, Bruno Schivinski, Wai Chen

**Affiliations:** 1 School of Science, Psychology and Sport, Federation University, Ballarat, Australia; 2 The Institute for Health and Sport, Victoria University, Melbourne, Australia; 3 Department of Psychology, University of Athens, Athens, Greece; 4 School of Media and Communication, Royal Melbourne Institute of Technology, Melbourne, Australia; 5 The Fiona Stanley Hospital, Youth Mental Health Unit, Murdoch, Perth, Australia; 6 Medical School, Curtin University, Perth, Australia; 7 Graduate School of Education, University of Western Australia, Perth, Australia

**Keywords:** Internet gaming disorder symptoms, Adults, Network analysis, Motivation

## Abstract

The study used regularized partial correlation network analysis (EBICglasso) to examine the structure of DSM-5 internet gaming disorder (IGD) symptoms (network 1); and the associations of the IGD symptoms in the network with different types of motivation as defined in the self-determination theory i.e., intrinsic motivation (engaging in an activity for something unrelated to the activity), identified regulation (engaging in the activity because it aligns with one’s values and/or goals), external regulation (engagement in activity being driven by external rewards and/or approval), and amotivation (engaging in an activity without often understanding why) (network 2). Participants were 968 adults from the general community. They completed self-rating questionnaires covering IGD symptoms and different types of motivation. The findings for network 1 showed mostly positive connections between the symptoms within the IGD network. The most central symptom was loss of control, followed by continuation, withdrawal symptoms, and tolerance. In general, these symptoms were more strongly connected with each other than with the rest of the IGD symptoms. The findings for network 2 showed that the different types of motivation were connected differently with the different IGD symptoms. For instance, the likeliest motivation for the preoccupation and escape symptoms is intrinsic motivation, and for negative consequences, it is low identified regulation. Overall, the findings showed a novel understanding of the structure of the IGD symptoms, and the motivations underlying them. The clinical implications of the findings for assessment and treatment of IGD are discussed.

**Supplementary Information:**

The online version contains supplementary material available at 10.1186/s12888-022-03708-6.

## Introduction

The fifth edition of the Diagnostic and Statistical Manual of Mental Disorders (DSM-5 American Psychiatric Association [APA], 2013 [1]) has included Internet Gaming Disorder (IGD) as a tentative disorder requiring further examination to be fully recognized as a bona fide diagnosis. In 2019, the World Health Organization (WHO) also recognized Gaming Disorder (GD) as a formal diagnosis in the 11th edition of the International Classification of Diseases (ICD-11; WHO, 2019 [2]). Given the earlier publication of DSM-5, numerous studies have adopted its proposed nine IGD symptoms for testing the structure and correlates of IGD (Gomez et al., 2019 [3]; Montag et al., 2019 [4]; Stavropoulos et al., 2018 [5]). Overall, findings to date using latent variable models have confirmed: a) the unidimensional structure of IGD via latent variable modelling studies and b) differential item/criterion functioning, considering discrimination and difficulty, via the use of item response theory evaluation (Király et al., 2017 [6]; Schivinski et al., 2018 [7]; Stavropoulos et al., 2019 [8]). However, no study to date has explored the interrelations between the nine DSM-5 suggested IGD symptoms, which concurrently embrace the component model of addictions (i.e., salience, tolerance, withdrawal, relapse, conflicts/ functional impairment and mood-modification; Griffiths, 2005 [9]). This is important as ICD-11 (WHO, 2019 [2]) conceptualized GD based on a number of only four criteria (i.e., impaired control over gaming; increased priority given to gaming; continuation despite negative consequences; experience of significant life problems due to gaming; Pontes et al., 2021 [10]). In that context, and in the light of the prospective DSM-5 revision and the potential inclusion of IGD as a formal diagnosis, the specific associations between the nine IGD criteria and their relevance for IGD need to be also investigated. This is imperative if their number of IGD symptoms is to be reduced without compromising the conceptualization and structure of this disorder. To address this need and extend this line of research, the current study used a novel emerging approach, called network analysis (Epskamp et al., 2018 [11]).

### Conceptualization and structure of IGD

According to the DSM-5 (APA, 2013 [1]), IGD is viewed as an excessive activity involving the persistent and recurrent internet use to play videogames, resulting to considerable impairment or distress over a period of 1 year. It has nine symptoms involving preoccupation with gaming; withdrawal symptoms when gaming is removed; tolerance or need to spend more time in gaming; unsuccessful attempts to control engaging in gaming; loss of interest in other activities because of gaming; continuation with gaming despite knowledge of problems; deception of other about the amount of time spend on gaming; using games to escape negative moods; and experience negative consequences, such as giving up relationships/opportunities because of gaming. Diagnosis requires at least five of these symptoms together with significant impairment over a period of 12 months.

To date, the structure of IGD symptoms has been examined extensively using statistical methods such as confirmatory factor analysis (CFA) latent class analysis (LCA), and less frequently, item response theory (IRT) models (Schivinski et al., 2018 [7]; Gomez et al., 2019 [3]; Stavropoulos et al., 2021 [12]), and conditional inference tree (Ctree) (Pontes et al., 2019 [13]). CFA studies have generally supported a one-factor IGD model (e.g., Pontes & Griffiths, 2016 [14]; Pontes, Stavropoulos, & Griffiths, 2017 [15]), and LCA studies have suggested the existence of a number of IGD typologies (Carras & Kardefelt-Winther, 2018 [16], Pontes et al., 2014 [17]). For example, Pontes et al. (2014 [17]) suggested five IGD typologies defined as “casual”, “regular”, “low risk”, “high risk” and “disordered gamers”. IRT studies have revealed variable discrimination, difficulty and reliability of the different IGD criteria (Gomez et al., 2019 [3]; Schivinski et al., 2018 [7]). More recently, Stavropoulos, Gomez, and Griffiths (2021 [12]) examined the structure of the IGD symptoms using factor mixture modeling (FMM). Although the findings showed most support for a model with two classes and one factor, there was also good support for the one-factor CFA model, and an LCA model with three classes (high, intermediate, and low endorsement classes). Considering IRT findings, different studies converged on the higher discrimination power of “withdrawal” compared to other IGD symptoms (Gomez et al., 2019 [3]; Schivinski et al., 2018 [7]). A common feature of these CFA, LCA, FMM, and IRT models is that they are all latent variable models (although adopting different calculation methods; Gomez et al., 2019 [3]). As applied to a psychological disorder, a latent variable model assumes the existence of a latent (unobservable) construct (which is the disorder in question) that causes a range of observable responses (that are the symptoms of the disorder). In such a model, the responses to observed symptoms are considered as reflecting the individual’s position on the latent construct. Also, the indicators (symptoms in the case of a clinical disorder) are considered to have nothing in common after controlling for the latent construct (an assumption referred to as local independence). Latent variable models are reflective models. A reflective model suggests that the symptoms of a disorder are interchangeable and equally reflective of the latent construct for the disorder. Seen in the contest of IGD, the reflective view indicates that the IGD symptoms (such as preoccupation and withdrawal symptoms) are interchangeable and equally reflective of the IGD. However, there is evidence suggesting that IGD symptoms may not be interchangeable and equally reflective of the IGD (Pontes et al., 2019 [13]). For instance, individuals with different IGD symptoms profiles show different associations with external variables (Stavropoulos et al., 2021 [12]). In another recent study, Pontes et al., (2019 [13]) used a machine learning approach (Ctree), to illustrate that different IGD criteria have different clinical weighing. Their study further supported that each individual criterion presents specific diagnostic roles in the development of IGD. Additionally, van Rooij et al., (2017 [18]) have argued that IGD may be better conceptualized as a formative construct. In the formative framework, causality is reversed and the symptoms are thought to cause the focal construct (Edwards & Bagozzi, 2000 [19]). Taking these into consideration, it can be argued that the IGD symptoms may be alternatively view in terms of causal relations between symptoms.

### Novel network model for IGD

Although the latent variable model is currently the most dominant approach for understanding psychopathologies, there have been recent developments in the application of network models (Borsboom & Cramer, 2013 [20]; Borsboom, 2008 [21]). In general, the network approach to psychopathology assumes that a disorder reflects the co-occurring of symptoms (Borsboom & Cramer, 2013 [20]). Symptoms are understood as a causal system, interacting with each other in meaningful ways, resulting in the disorder (Borsboom & Cramer, 2013 [20]). As noted by Armour, et al., (2017 [22]), symptoms by their very nature have direct relations to one another, and therefore the idea that symptoms do not interact with each other causally is highly implausible.

Overall, the focus of networks is on the relationships among the symptoms of the disorder (Borsboom & Cramer, 2013 [20]). Network model can be tested empirically using network analysis (Borsboom & Cramer, 2013 [20]; Boschloo et al., 2015 [23]). Network analysis is an exploratory approach that provides quantitative and visual information about symptoms that are core or central (importance) to the overall network of symptoms, and the connections between symptoms (Borsboom & Cramer, 2013 [20]; Fried et al., 2015 [24]). In network analysis, one creates a network based on partial correlations between variables. As noted by Epskamp and others (Epskamp & Fried, 2018 [25]; Epskamp et al., 2017 [26]), such a network can identify unique interactions between variables that cannot be identified using multiple regression analysis, and when the network analysis is exploratory it is advantageous over structural equation modelling, because there are no equivalent undirected models possible. Network models can also include other variables (external correlates), and thereby examine how the different symptoms of a disorder are linked with the external correlates. More details on network analysis are provided in the Methodology section.

### IGD and motivation

To date many studies have examined the relations between motivation and internet gaming disorder (e.g., Beranuy et al., 2013 [27]; Hussain et al., 2015 [28]). Generally, these studies have shown that IGD is predicted by motivations related to achievement, socialization, and escape from the real world (Carlisle, 2017 [29]). The motivation for IGD had also been examined (Mills & Allen, 2020 [30]; Mills et al., 2018 [31]) in terms of the Self-Determination Theory (SDT; Deci & Ryan, 1985 [32], 2000 [33]), which is arguably a major theory of human motivation.

SDT (Deci & Ryan, 1985 [32], 2000 [33]) is arguably a major theory of human motivation. According to this theory, different types of motivation (quality) determine different behaviors. These types of motivation differ in their inherent levels of self-determination that in seen as being on a continuum from high to low autonomy, hence, intrinsic motivation, extrinsic motivation, and amotivation. Intrinsic motivation refers to engaging in an activity for the pleasure and enjoyment in the activity itself. Extrinsic motivation refers to something unrelated to the activity (e.g., wanting to becomes more socially accepted) as facilitating engagement. To date, four subtypes of extrinsic motivation have been discussed: external regulation, introjected regulation, identified regulation, and integrated regulation. In external regulation, activity engagement is seen as been driven by external rewards and/or approval. Introjected regulation involves unsanctioned internal pressures (e.g., subjective obligation driving the engagement in the activity). Identified regulation refers to individuals engaging in the activity because it aligns with their values and/or goals. Integrated regulation occurs when one’s engagement is driven from a belief that one’s self is expressed through it. Amotivation involves engaging in an activity without often understanding why (e.g., one presents rather unwilling to engage with an activity).

In addition to self-determination, SDT postulates that individuals are motivated to behave in ways that fulfill three basic psychological needs, those needs being competence (need for being effective in one’s interactions with the environment), autonomy (a sense of feeling free from pressures and to have the possibility to make choices), and relatedness (fundamental striving for contact with others.) Importantly, a recent meta-analysis showed that integrated regulation is strongly associated with both identified regulation and intrinsic motivation, and therefore cautioned against concurrently measuring it, due to collinearity (Howard et al., 2017 [34]). According to STD, context that offers opportunities to satisfy basic needs will lead to high self-determined types of motivation, such as intrinsic motivation and identified regulation. In contrast, context that thwart these needs will lead to low self-determined types of motivation, such as external regulation and amotivation.

Empirical data indicates the intrinsic, integrated and identified regulation types of motivation are associated positively with adaptive outcomes (e.g., positive for one’s wellbeing and development), whereas, introjected, external regulation, and amotivation are associated with maladaptive outcomes (e.g., Clarke, 2004 [35]; Litalien et al., 2015 [36]; Mouratidis et al., 2011 [37]). This has also been replicated in the context of video gaming (Peracchia et al., 2019 [38]). Indeed, there is now STD-based data showing that extrinsic motivations (introjected regulation more than other types of extrinsic motivations) and intrinsic motivation are associated with more IGD engagement, compared to amotivations (Mills & Allen, 2020 [30]; Mills et al., 2018 [31]). Furthermore, there is evidence indicating the usefulness of applying SDT to predict a range of behaviors. Of relevance to the current study, existing data has shown that low self-determination in gambling-related or exercise activities predicted greater gambling disorder and exercise dependence (both being considered to be addictions), respectively (Clarke, 2004 [35]; González-Cutre & Sicilia, 2012 [39]). Given this, it can be speculated that STD could have merits in explaining gaming behaviors and disorders (also considered an addiction). Therefore, it is conceivable that different types of motivations may underpin the different IGD symptoms, and that these associations can be revealed by conducting a network analysis that includes both the IGD symptoms and different types of motivation. To the best of our knowledge, no study to date has adopted this approach to address the whole range of IGD behaviors.

### Clinical importance of network analysis

Results from network analysis can have important implications for theory, assessment and diagnosis, treatment and prevention, and understanding comorbidity. It is important to note that symptom centrality and symptom mean are different as they are often weakly associated (Yang et al., 2016 [40]), i.e., the mean levels of symptoms can change without changes in its centrality in network. Thus, different conclusions about what is core symptom in a disorder could be arrived at when looking at symptom centrality and symptom severity (Mullarkey et al., 2019 [41]). As symptoms for a disorder identified as central in a network are considered most influential in producing or maintaining the disorder, intervening on these symptoms can be expected to maximize the impact of intervention. In this respect, and given its network characteristic, focusing on the central symptoms could potentially have a downstream effect in improving other symptoms. The inclusion of external correlates to a network reflecting a disorder could provide useful information for theory and treatment. For instances, with reference to motivation, if we know how the central IGD symptoms are associated with the different types of motivations, we could be able to design better targeted interventions, and thereby expect faster and better outcome. Despite the noted advantages of the network approach, to the best of our knowledge, to date, no study has examined IGD symptoms using network analysis or the associations of the IGD symptoms on their own, or with different types of motivation.

However, it may be worth noting that other approaches have also been used to ascertain the central IGD symptoms, for example, Lee et al. (2017 [42]) examined the distribution of the ICG symptoms in different IGD severity groups. They found that giving up other activities and negative consequences, and to a lesser degree continuation indicated more severity of IGD, with tolerance not been influenced by IGD severity. Ko et al. (2020 [43]) found that except for deception and escape, all the other symptoms had high diagnostic accuracy for differentiating adults with and without internet gaming disorder. Based on item response theory, Schivinski et al. (2018 [7]) found that criteria for continuation, deception, and escape presented a poor fit to the model and had notable higher difficulty parameter values. Similar results were later reported by Gomez et al. (2019 [3]), who found that the symptoms for negative consequences, deception, and continuation in a different sample also presented higher difficulty parameter values, thereby indicating that these symptoms measure more severity.

### Aims of the present study

Based on the aforementioned literature, there were two major aims in the current study. Firstly, the study used network analysis, with regularized partial correlation, to examine the network structure of the nine DSM-5 IGD symptoms (preoccupation, withdrawal, tolerance, unsuccessful attempts to control usage, loss of interest in other activities, functional impairment, deception, escaping reality, and mood-relief in relation to Internet games’ usage), in a large online community sample. We produced a network graph; evaluated the symptoms and the associations between the symptoms most influential (edge weight and centrality) in the network; and the robustness and stability of the network. Second, we used network analysis to examine how specific OIT motivation types (intrinsic motivation, identified regulation, external regulation, and amotivation) are linked with the different IGD symptoms.

## Method

### Participants

Participants were from the general community. This was a normative online convenience sample, comprising 968 English speaking adults. In terms of power for network analysis, a sample size of at least 250 participants has been suggested for accurate and reliable network analysis results (Epskamp & Fried, 2018 [25]). Thus, the current sample size of 968 participants provided sufficient power for the study. The age of participants ranged from 18 to 64 years (mean = 29.54 years; *SD* = 9.35 years), and included 622 males (64.9%; mean age = 29.46 years, *SD* = 8.93 years), and 315 females (32.9%; mean age = 30.02 years, *SD* = 10.39 years). Additionally, 26 individuals (2.7%) identified themselves as trans/non-binary gender, 1 individual identified as queer without further specification, 1 individual as 'other', and 3 individuals did not specify their gender. No significant age differences were found across males and females, *t* (935) = 0.846, *p* = 0.398.

Supplementary Table S1 presents the sociodemographic and internet games use information for the sample. In terms of sociodemographic background, slightly more than half the number of participants reported being employed (55%; *n* = 532) and most of them reported having completed at least secondary education (98.2%; *n* = 12 out of 668 scores). In terms of internet gaming, on average, respondents spent an average of approximately 9.48 h per week playing video-games. Considering IGD, 66 participants (6.8%) exceeded the cut-off diagnostic threshold for the IGDS9-SF proposed by Pontes and Griffiths (2016 [14]; see Method section). However, in terms of cut-off score for determining IGD, a recent study found that a cut-off of 32 may be adequate to distinguish between disordered and non-disordered gamers (Qin et al., 2020 [44]). Based on this cut-off score, only 49 participants (5.1%) of the sample in the study would be considered to have problematic IGD symptoms. Overall, therefore, the sample comprised mainly online employed male gamers, with non-pathological levels of gaming engagement.

### Measures

The measures included questions seeking sociodemographic (i.e. age, gender, education, occupation, relationship status etc.), internet gaming information (being involved with internet gaming, number of years engaged in internet gaming, and hours spent on weekdays and weekends in internet gaming), disordered gaming (Pontes & Griffiths, 2015 [45]), and motivation (Guay et al., 2000 [46]).

*Internet Gaming Disorder Scale – Short-Form (IGDS9-SF;* Pontes & Griffiths, 2015 [45]). The IGDS9-SF was used to assess DSM-5 (APA, 2013 [1]) IGD symptoms. The IGDS9-SF is arguably the most broadly utilized scale currently in the field of disordered gaming (Stavropoulos et al., 2021 [12]). The IGDS9-SF includes the nine DSM-5 symptoms with a time reference of the past year. An example item is: “Do you feel more irritability, anxiety or even sadness when you try to either reduce or stop your gaming activity?” Items are responded to on a five-point scale from 0 (‘Never’) to 4 (‘Very often’). Therefore, higher symptom scores indicating higher symptom severity. Overall, given its good psychometric properties, its alignment with the symptoms for IGD in DSM-5, and its wide acceptance, the IGDS9-SF can be considered useful for IGD research studies (Pontes & Griffiths, 2015 [45]), including examination of the IGD network structure. The internal reliability for the IGDS9-SF instrument was very good in the present study (Cronbach α = 0.88). Pontes and Griffiths, (2016 [14]; see also Pontes et al., 2016 [47]) advocated that meeting five or more of the nine the IGDS9-SF items, based on ‘often’ and ‘very often’ responses, provides a diagnostic indication in line with the DSM-5 proposals.

*Situational Intrinsic Motivation Scale (SIMS*; Guay et al., 2020 [46]). The SIMS is a 16-item scale, with four dimensions, related to different types of motivation described in the Self- Determination Theory (SDT; Deci & Ryan, 1985 [32], 1991 [48]). The dimensions are intrinsic motivation, identified regulation, external regulation, and amotivation. An example of intrinsic motivation is “Because I think that this activity is interesting”; identified regulation is “Because it’s for my own good”; external regulation is “Why am I supposed to do it”; and amotivation is “I don’t know; I don’t see what this activity brings me”. For all items, participants are asked to respond to the stem, “Why are you currently engaged in this activity?” For the SIMS, participants respond to each item using a 7-point scale, ranging from 1 (does not correspond at all) to 7 (corresponds exactly) for an activity. Higher scores indicate greater self-determination towards the situation being analyzed. For the current study individuals were asked to respond to the questions with internet gaming as the reference activity. The SIMS and its dimensions have shown sound psychometric properties, including factor structure, reliability and validity (Guay et al. 2000 [46]). The internal reliability (Cronbach α) for the intrinsic motivation, identified regulation, external regulation, and amotivation subscales in the present study were α = 0.87; α = 0.76; α = 0.82, and α = 0.81, respectively.

### Procedure

The study was approved by the Human Ethics Research Committee, Victoria University (Australia). The study was advertised using both nonelectronic and electronic (i.e. email, social media) methods. Due to the inclusion of questionnaires addressing one’s level of distress, those who had a current untreated severe mental illness were instructed (also included in the plain language information statement) not to participate so as to avoid any unforeseen/ indirect emotional impact. The survey was conducted online. Participants were invited to register into the study via a Qualtrics link available on social media (i.e. Facebook; Instagram; Twitter), the Victoria University websites and digital forums (i.e., reddit.com). The link took them to the Plain Language Information Statement (PLIS). Those wishing to participate were directed to click a button to agree to informed consent. This was followed by the questions seeking sociodemographic and internet gaming information, and then the SIMS (Guay et al., 2000 [46]) and IGDS9SF (Pontes & Griffiths, 2015 [45]) questionnaires. Participants completed the online survey using a computer in a location of their choosing.

### Statistical network analyses

Given the aims of the study, we computed two network models: a network model with only the nine IGD symptoms present in the IGDS9-SF (network 1), and a network model with the nine IGD symptoms present in the IGDS9-SF and the four domains in the SIMS (network 2). Missing data was handled using the “exclude pairwise method”. In network analysis, variables are referred to as nodes, and the relationships between the nodes are referred to as edges. The strength of the relationship between nodes is indicated in terms of edge weights. Network nodes and edges can be estimated using zero-order correlations. In such instances, the edges between nodes will not control for the relations with other nodes, thus inflating correlations, and therefore resulting in difficult to interpret and misleading results. To overcome this, a regularized partial correlation approach, such as the graphical Least Absolute Shrinkage and Selection Operator (g-lasso; Tibshirani, 1996 [49]) is used to compute network analysis. Lasso shrinks small partial correlations to 0, resulting in a sparse network, and showing only the most important relationships in it. When a lasso-based approach is applied, there is generally low likelihood of false positives, thereby providing confidence of edges reported in the network (Krämer et al., 2009 [50]). However, g-lasso can result false negatives, and therefore the absence of an edge between two nodes cannot be automatically assumed to mean no relation between them. The bootnet (Epskamp et al., 2018 [11]) and the qgraph (Epskamp et al., 2012 [51]) packages from R are used to conduct network analyses and network graphs, respectively.

For the current study, for both networks 1 and 2, we used the network module provided in Jeffreys’ Amazing Statistics Program (JASP) version 0.14.1.0 statistical software (Team JASP, 2018 [52]). We applied the extended Bayesian information criterion (EBIC) glasso for the network analysis, as used in other studies (Heeren et al., 2018 [53]; Isvoranu et al., 2017 [54]; McNally et al., 2017 [55]). The EBICglasso produces the optimal degree of shrinkage according to an EBIC and a hyperparameter. The hyperparameter in the study was set at 0.5, since it is suggested to produce networks that balance specificity and interpretability with sensitivity (Foygel & Drton, 2010 [56]; Epskamp & Fried, 2018 [25]). Consequently, the network produced a model that is sparser and easier to interpret, with blue edges indicating positive relations, and red edges indicating negative relations. Additionally, edge weights (strength of the relation between nodes) are shown in terms of varying the thickness and color density of the edge connecting the nodes, with thicker denser colored lines indicating stronger relationships. The distance between nodes is indicative of the relationship between them (i.e., nodes with stronger similarities are close together) Also, as the qgraph package applies the Fruchterman and Reingold’s (1991 [57]) algorithm to position the nodes, nodes with stronger correlations are positioned near the center of the network, while nodes with weaker correlations are positioned near the periphery of the network.

Apart from visualization of the network graph, the network can be described statistically in terms of edge weights and centrality of the nodes (Borgatti, 2005 [58]). An edge weight indicates the strength of the relationship between nodes. For the edge weights, minimum absolute value of 0.03 was considered as worthy of interpretation (Isvoranu et al., 2017 [54]), Centrality refers to the relative importance of the individual nodes in the network. A central symptom is one that is highly connected to other symptoms, and its activation can be expected to spread to other symptoms. In contrast, a symptom with low centrality has fewer connections with other symptoms, and has less influence on the network. Common reported indices of centrality are strength, betweenness, closeness, degree, and expected influence (Opsahl et al. 2010 [59]). Betweenness of a node is the number of times a symptom lies along the shortest paths between other pairs of symptoms, with a value of zero suggests that the node is not present on the shortest pathway between them, and high betweenness centrality values indicate that they highly present on the shortest pathway between them. Closeness is the inverse of the sums the shortest path lengths between a given node and all other nodes in the network. Thus, nodes with high closeness centrality values indicate that they are central in network (connecting with other nodes). Degree (called strength in a weighted network, as is the case in the current study) is the sum of all direct associations a given symptom exhibits with all other nodes; and it reflects the direct influence a given node has on the network. Again, nodes with high degree centrality values indicate that they are more central. The expected influence for a node is the absolute sum of edge weights associated with it, taking into consideration negative nodes. Of these centrality indices, expected influence been developed primarily for psychological networks (Robinaugh et al., 2016 [60]), and it is less prone to the interpretive challenges present in the other centrality indices (Bringmann et al., 2019 [61]). Taking these into consideration, although we report the centrality indices for strength, betweenness, closeness, degree (strength), and expected influence, we focused on expected influence for ascertaining centrality of the nodes in the study.

A network must also be evaluated for its accuracy and stability. Network accuracy and stability refer to the likelihood that the network results will be replicated. For this, it has been recommended that the accuracy of the edge and the stability of the centrality estimates should be examined. One way to estimate the accuracy of edge weights is using bootstrap 95% non-parametric confidence intervals (CIs) (Epskamp et al., 2018 [11]). Narrower CIs suggest a more precise estimation of the edge (Epskamp et al., 2018 [11]). Expressed differently, if the CIs around most of the estimated edge-weights are large, it means that many of the edge-weights are likely not to differ significantly from one-another, and therefore interpreting the order of most edges in the network could be problematic, and has to be done with care. The stability of the centrality indices can be examined by using a different type of bootstrapping referred to as case-dropping (or alternatively node-dropping) bootstrapping (Epskamp & Fried, 2018 [25]). This procedure examines if the order of centrality indices remains the same after re-estimating the network with less cases (or nodes). It quantifies the stability of centrality indices in terms of correlation stability coefficient. This coefficient reflects the correlation between the original centrality indices (based on the full data) and the correlation obtained from the subset of data representing different percentages of the overall sample. Although a correlation stability coefficient of 0.7 or higher has been suggested as being the threshold, Epskamp et al. (2018 [11]) have suggested that the correlation stability coefficient should not be below 0.25, and preferably it should be above 0.5. For the current study, the stability of the centrality indices and edge accuracy of the network were examined using the procedures just described. Both were estimated with 1000 bootstraps. Also, as the correlation stability coefficient was not available for expected influence with JASP (Team JASp, 2018 [52]), the software used in the current study to compute network analysis, we focused on the accuracy of the other centrality indices.

## Results

### Descriptive information of data

Initially we examined the mean and standard deviation (*SD*) scores. The findings are presented in Supplementary Table S2. As show, the mean score for the nine symptoms ranged from 1.38 (negative consequences) to 3.05 (escape), thereby indicating wide variability.

### Network 1: network analysis of the IGDS9-SF symptoms

#### Visualization of the IGD network

**Fig. 1:**
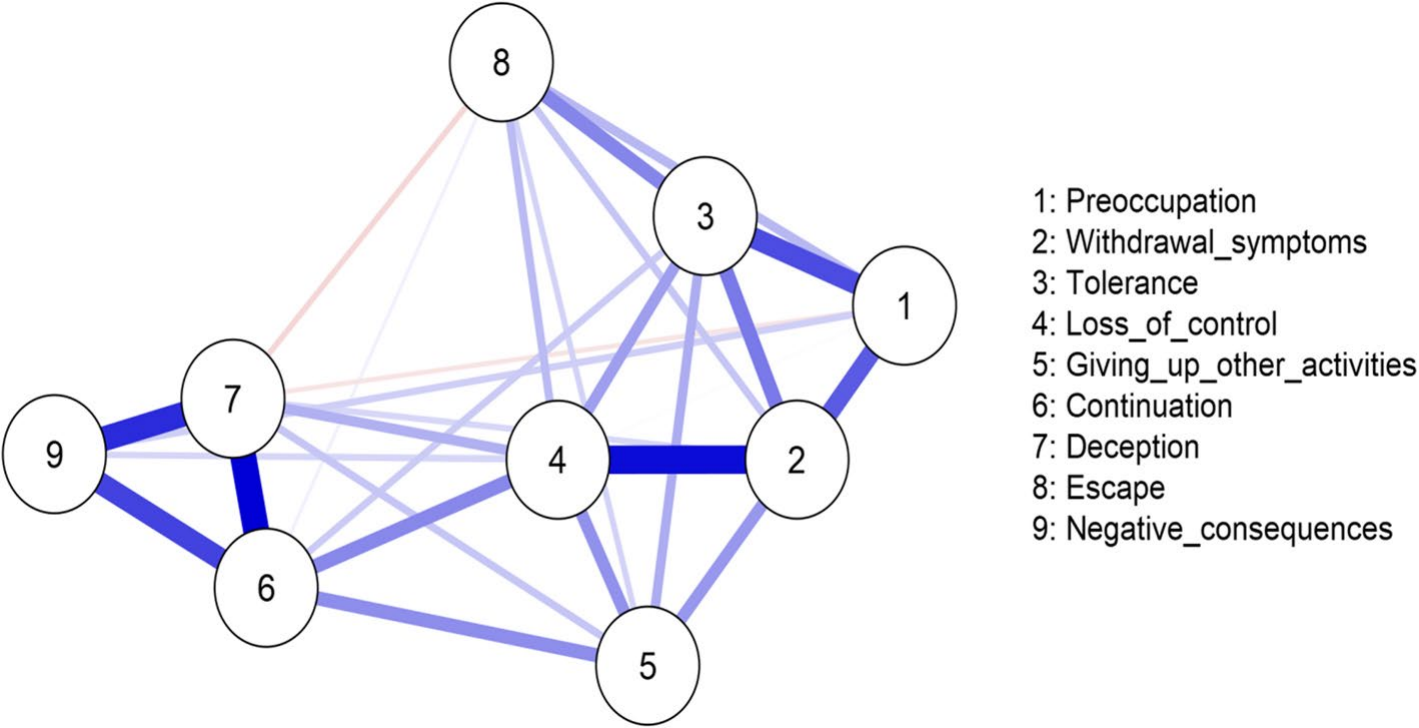
Network of the IGDS9-SF variables. Blue lines represent positive associations, and red lines negative associations. The thickness and brightness of an edge indicate the association strength. The layout is based on the Fruchterman–Reingold algorithm that places the nodes with stronger and/or more connections closer together and the most central nodes into the center

With nine nodes, the maximum number of edges in this network was 36. However, the EBICglasso estimation used in the analysis reduced the number of edges that were estimated to 29. Figure 1 shows a visualization of the network of the nine IGD symptoms in the IGDS9-SF. As shown in Fig. 1, except for deception (7) with preoccupation (1) and with negative consequences (8), all other nodes were associated positively (blue edges) with one another. Also, loss of control (4) was placed in the center of the network and had more connections with the rest of the nodes in the network.

#### Edge weight of IGD symptoms in the IGD network

Supplementary Table S3 shows that weights matrix between the IGDS9-SF nodes from the network analysis. As shown in this table (see also Fig. 1), in decreasing order, the edges were especially strong between continuation (6) and deception (7), loss of control (4) and withdrawal (2), deception (7) and negative consequences (9), negative consequences (9) and continuation (6), preoccupation (1) and tolerance (3), and withdrawal (2) and preoccupation (1). There was no connectivity between the nodes for continuation (6) with preoccupation (1) and withdrawal symptoms (2); deception (7) and tolerance (3); and negative consequences (9) with tolerance (3), giving up other activities (5) and escape (8).

**Fig. 2:**
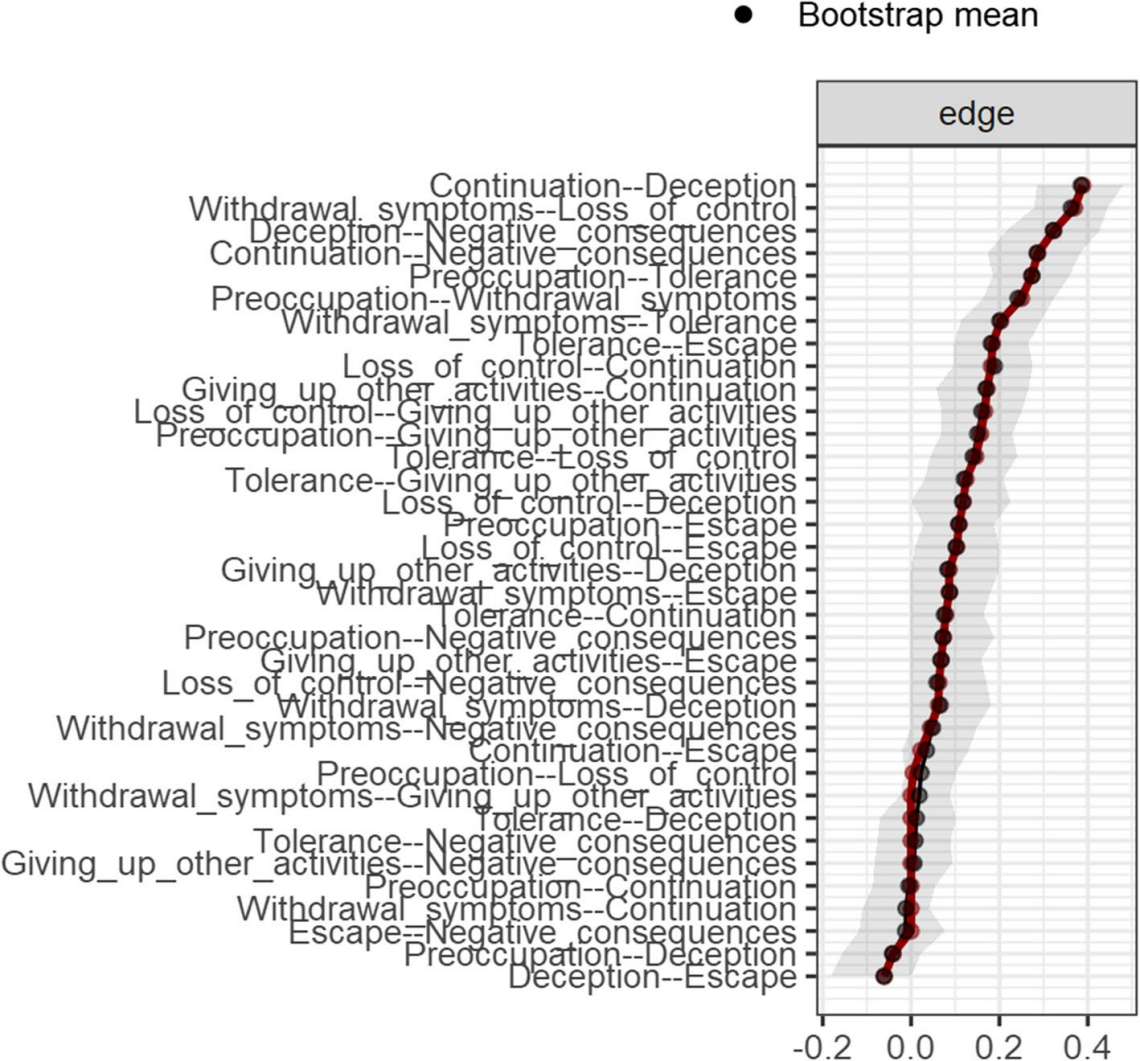
Edge stability estimate using non-parametric bootstrapped estimate. The x-axis represents the edges, while every line on the y-axis represents a specific edge. The red line shows the estimate of the edge stability, and the gray bars the 95% confidence intervals (grey bars) for the estimates

The accuracy of the edge weights, estimated using bootstrap 95% non-parametric CIs is shown in Fig. 2. As shown, although the 95% CI of most of the edges included zero, some of the 95% CI of the edges did not include zero. Also, the CIs around a large number of the estimated edge-weights were large, thereby indicating that many of the edge-weights did not differ significantly from each other, and therefore the interpretation of the order of many edges in the network is problematic, and needs to be viewed cautiously.

#### Centrality of the IGD symptoms in the IGD network

**Fig. 3:**
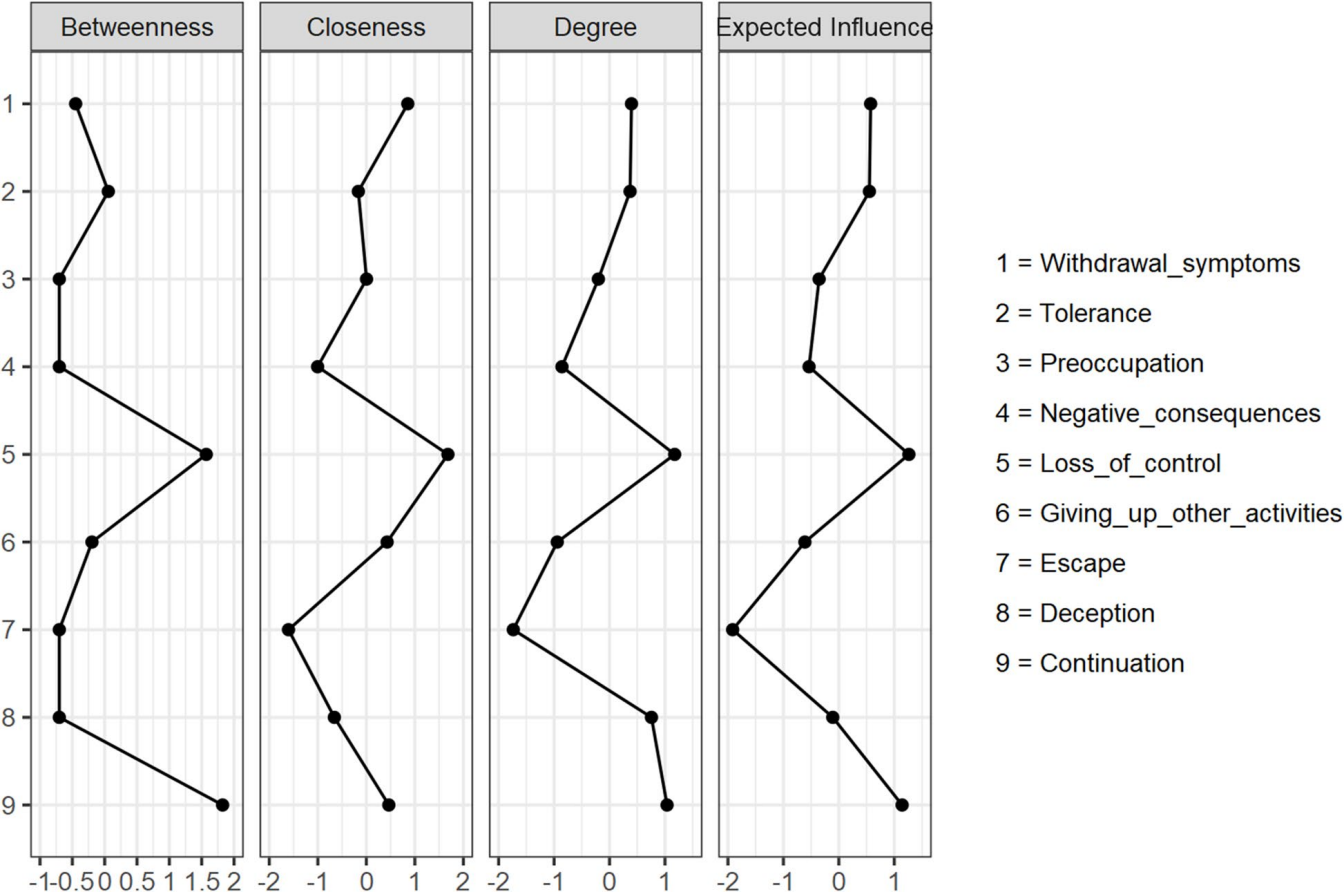
Centrality plots for the association in the network of each node in standardized z values. Values shown on the x-axis are standardized z-scores. Symptom numbers in the IGDS9-SF are as follows: Withdrawal symptoms = 2; tolerance = 3; preoccupation = 1; negative consequences = 9; loss of control = 4; giving up other activities = 5; escape = 8; deception = 7; continuation = 6

The standardized estimates of the centrality indices for betweenness, closeness, strength, and expected influence are presented in Table 1. To ease interpretation, plots for the centrality measures in terms of *z* scores were created, and this is displayed in Fig. 3. Both Table 1 and Fig. 3 present the centrality indices for betweenness, closeness, and degree (strength). Table 1 also reports the expected influence values, but this is not show in Fig. 3 as this is not available in JASP (Team JASP, 2018 [52]), the software used in the current study to compute network analysis, As shown in Table 1 and Fig. 3, for the different nodes, there was notable variability in their relative values. Thus, to ensure clear interpretation of centrality, we examined the results from the analysis involving test of the accuracy for the centrality indices, and then selected the index deemed most accurate.

**Table 1:** Centrality indices of IGDS9-SF variable from the network analysis

IGDS9-SF variable	Betweenness	Closeness	Strength	Expected influence
1. Preoccupation	−0.701	0.004	−0.201	− 0.356
2. Withdrawal symptoms	−0.449	0.854	0.396	0.574
3. Tolerance	0.056	−0.165	0.371	0.551
4. Loss of control	1.571	1.682	1.175	1.263
5. Giving up other activities	−0.196	0.428	−0.943	− 0.612
6. Continuation	1.823	0.464	1.038	1.141
7 Deception	−0.701	−0.659	0.756	−0.110
8. Escape	−0.701	−1.604	−1.735	−1.915
9. Negative consequences	−0.701	−1.004	−0.858	− 0.537

Higher numbers indicate that the variable is more central to the network; highest two values are underlined within each index

**Fig. 4:**
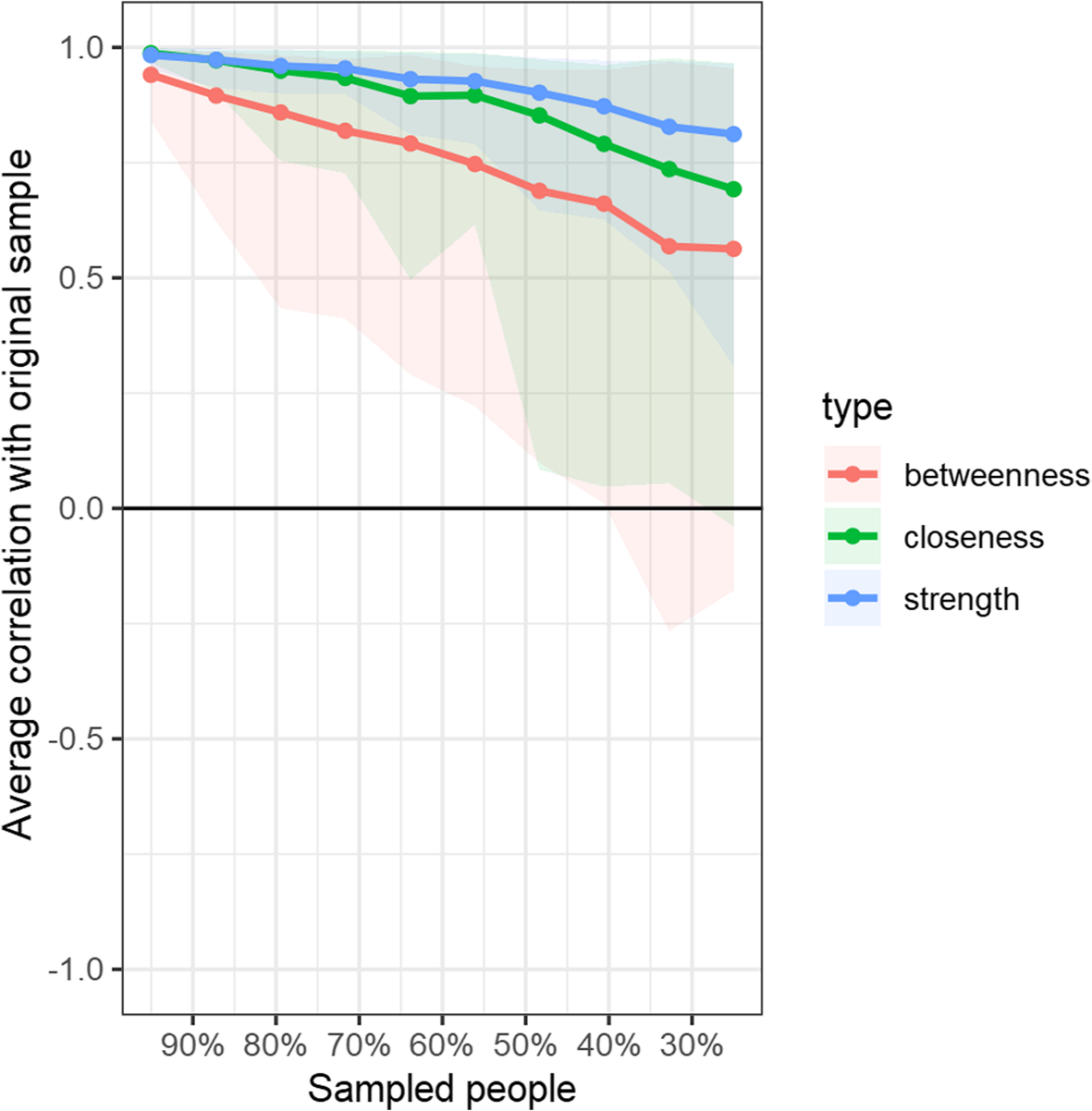
Stability of central indices

The stability of the centrality indices (betweenness, closeness, and strength) examined using case-dropping bootstrapping is shown in Fig. 4. The figure shows that for all centrality indices, the correlation stability (CS) coefficient from the subset of data representing different percentages of the overall sample. Figure 4 shows that there was a slight drop in the correlations between the subsample estimate and the estimate from the original entire sample as the subset samples decreased from 95% of the original sample to 25% of the sample. However, for this, the correlations for all the centrality indices (betweenness, closeness, and strength) remained above 0.7, thereby indicating stability for the centrality indices (Epskamp et al., 2018 [11]). Notwithstanding this, for the current study we focused on strength centrality as it is known to reflect reasonably precise centrality estimates for psychology networks (Santos et al., 2018 [62]).

In general, for all centrality indices, including expected influence, higher values indicate more centrality. As shown in Table 1, the two nodes (in descending sequence) with the highest strength centrality values were loss of control (4), continuation (4). As shown in Fig. 1, these nodes had relatively many and strong connections. The two nodes with the lowest strength centrality values were escape (8), and giving up other activities (5). As shown in Fig. 1, these nodes had relatively few and weak connections.

### Network 2: combined IGD and situational motivation scale (SIMS) dimensions network

**Fig. 5:**
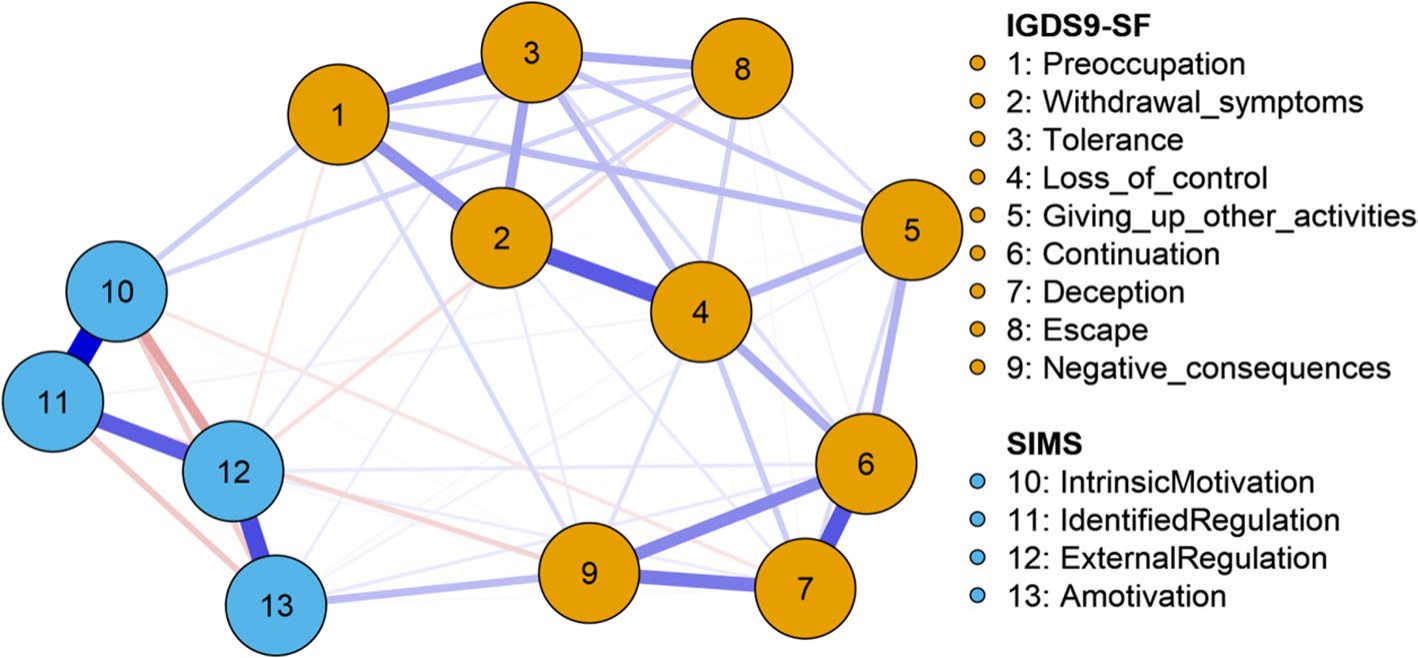
Network of the IGDS9-SF variables together with the Situational Motivation Scale (SIMS)

Figure 5 shows a visualization of the network of the nine IGD symptoms in the IGDS9-SF together with the four motivation dimensions included in the SIMS. It will be noticed that generally the inclusion of the motivation variables had little impact on the IGD network. Also, the connectivity between the motivation variables was generally stronger than between them and the IGD symptoms.

For the motivation dimensions, the edges for nodes intrinsic motivation (10) and identified regulation (11), identified regulation (11) and external regulation (12), and external regulation (12) and amotivation (13) were positive (blue edges in Fig. 5). The edge weights were 0.57, 0.36, and 0.40, respectively. The edges for intrinsic motivation (10) and external regulation (12), intrinsic motivation (10) and amotivation (13), and identified regulation (11) and amotivation (13) were negative (red edges in Fig. 5), with edge weights − 0.202, − 0.112, and − 0.120, respectively. The strength centrality indices for intrinsic motivation (10), identified regulation (11), external regulation (12), and amotivation (13) were 0.757, 0.866, 1.298, and − 0.802. Thus, apart from amotivation, the other motivation types (external regulation, identified regulation, and intrinsic motivation) can be considered central motivation nodes in the combined IGD-motivation network.

As shown in Fig. 5 and Table S4, preoccupation (1) was associated positively with intrinsic motivation (0.109) and negatively with external regulation (− 0.047). Withdrawal symptoms (2) was associated positively with both external motivation (0.006) and amotivation (0.037). Tolerance (3) was associated positively with external motivation (0.049). Loss of control (4) was also associated positively with both identified regulation (0.049) and amotivation (0.014). Giving up other activities (5) had negative association with identified regulation (0.009), and positive association with amotivation (0.027). Continuation (6) had positive associations with external regulation (0.050) and amotivation (0.045). Deception (7) was associated negatively with intrinsic motivation (−.058), and positively with external regulation (0.039) and amotivation (0.012). Escape (8) was associated positively with intrinsic motivation (0.095) and negatively with external regulation (− 0.071); and negative consequences (9) was associated negatively with intrinsic motivation (− 0.012) and identified regulation (− 0.093), and positively with external regulation (0.016) and amotivation (0.149).

## Discussion

The current study is the first to use network analysis to examine the structure of the nine DSM-5 IGD symptoms (APA, 2013 [1]). This was examined in an online normative-community sample. It examined the centrality of the IGD symptoms in the network; the edge weights for the IGD symptom pairs, the stability and accuracy of indices for centrality and edges; and how the IGD symptoms in this network were associated with intrinsic motivation, identified regulation, external regulation, and amotivation.

### IGD network: symptom centrality and associations between symptoms

The key findings from the network analysis were that there was no connectivity for continuation with preoccupation and withdrawal symptoms; deception and tolerance; and negative consequences with tolerance, giving up other activities, and escape. For symptoms with connectivity, they were generally positive. The exceptions were deception with preoccupation and negative consequences. The three highest edges were between continuation and deception, loss of control and withdrawal, deception and negative consequences. Loss of control was placed in the center of the network and had connections with all the other symptoms in the network, with especially stronger associations with withdrawal symptoms, tolerance, and continuation. Continuation and withdrawal also had relatively high centrality values. The two least central nodes were escape, and giving up other activities. These symptoms had relatively few and weak connections. In relation to the reliability of our findings, the study showed moderate support for edge weights, and strong support for centrality. Thus, although the strength centrality can be interpreted with some confidence, there is need for some caution when interpreting edge weights.

From a network perspective, symptoms with high centrality suggest that they are important. Also, the absence of a connection between two symptoms implies that they are conditionally independent of each other given the other symptoms in the network and maybe less important. Thus, our findings suggest that loss of control, continuation, and withdrawal are especially important and central for understanding IGD, with loss of control being most important. In contrast, escape, and giving up other activities did not present to be central to IGD experience.

### Clinical implications

Our findings have implications for theory, assessment and diagnosis, treatment and prevention, and understanding IGD comorbidity. Firstly, the absence of a connection between two symptoms in a network implies that they are conditionally independent of each other given the other symptoms in the network. As our findings showed no connectivity for continuation with preoccupation and withdrawal symptoms; deception and tolerance; and negative consequences with tolerance, giving up other activities, and escape, the symptoms in these relations can be considered as conditionally independent of each other. This is a novel finding, and indicates that there may be a need to reconsider the relevance of these symptoms for IGD. This conclusion is further reinforced by our findings that showed that deception was associated negatively with preoccupation and negative consequences.

Secondly, in a network, symptoms with high centrality values are considered most influential in producing or maintaining the disorder. Given that our findings showed that of the nine symptoms, loss of control, continuation, and withdrawal have the highest three centrality values, it can be argued that these three symptoms are more important than the other symptoms for understanding and diagnosis of IGD. Of these, loss of control showed to be most important. Thus, individuals with serious problems related to loss of control, continuation, and withdrawal are likely to demonstrate or to be at risk for more serious IGD presentations. This will be especially so for individuals with serious loss of control issues. Indeed, our findings suggest that an individual with loss of control problems is likely to show all the other eight symptoms. Thus, clinicians may wish to pay special attention to the presence and severity of this symptom and those related to continuation, and withdrawal during assessment and diagnosis of IGD.

Thirdly, because the symptoms with high centrality values are considered most influential, intervening on these symptoms could maximize the impact of an intervention, including reducing the effects of other symptoms. This, therefore, could mean that focusing intervention efforts on loss of control, continuation, and withdrawal rather than the other symptoms could maximize treatment effects, and also likely cascade to reduce the effects of other symptoms. Where relevant, focusing on the symptoms with high centrality values (loss of control, continuation, and withdrawal in the case of IGD) may also prevent the on-set and development of IGD in the context of primary prevention protocols implemented in the community.

### Associations between the IGD network nodes and types of motivation

In the combined IGD-motivation network model, the connectivity between the motivation variables was generally stronger than between them and the IGD symptoms. With reference to the centrality of the motivation nodes, external regulation, identified regulation, and intrinsic motivation had relatively high values. The value for amotivation was low and negative. In terms of the relations for the IGD symptoms with the motivation dimensions, there were many associations, and multiple associations for some IGD symptoms with different motivation types. Despite this, many of the associations were relatively low. If we consider values of around 0.03 and above as reasonably important edge values (Isvoranu et al., 2017 [54]), then the associations that are important in this network are preoccupation and escape, being associated positively with intrinsic motivation, negative consequences being associated positively with amotivation; and negative consequences being associated negatively with identified regulation; deception being associated negatively with intrinsic motivation; loss of control being associated positively with identified regulation; tolerance, continuation, and deception being associated positively with external regulation; preoccupation and escape being associated negatively with external regulation; and withdrawal symptoms and continuation being associated positively with amotivation. Of these the associations were especially string for preoccupation and escape with intrinsic motivation, negative consequences with amotivation; and negative consequences with identified regulation.

Viewed from a network perspective, the centrality findings indicate that external regulation, identified regulation, and intrinsic motivation (but not amotivation) can be considered important motivation types for understanding the motivations underpinning IGD. Also, the most likely reasons (motivation) for the behaviors reflecting preoccupation and escape are intrinsic motivation. For negative consequences, it is likely to be low identified regulation. Findings such as these suggest that IGD is driven primarily for the intrinsic values the games offer as well as values identified in these games by the player. This conclusion is in line with existing data showing that those extrinsic motivations (introjected regulation more than other extrinsic motivations) and intrinsic motivation more than amotivations are associated with greater IGD problems (Mills et al., 2020 [30]; Mills et al., 2018 [31]).

As we included different types of motivation in the second network model tested, we were able to show that external regulation, identified regulation, and intrinsic motivation (but not amotivation) are major motivations for IGD type engagement with video gaming. As will be recalled, in the SDT, intrinsic motivation refers to behaviors that are engaged in for their own sake, in terms of the pleasure and satisfaction derived from performing them. Identified regulation is a form of intrinsic motivation that refers to behaviors that are valued and perceived as being chosen by oneself – yet not performed for itself but as a means to an end. These findings are useful when planning interventions for those with IGD.

### Study limitations and directions for further studies

The results have to be interpreted in the light of a number of limitations. Firstly, as the study showed only moderate stability and accuracy for edge weights, the edge weight findings need to be interpreted with some caution. Secondly, network analysis assumes that mental disorders (and therefore IGD) are causal systems. However, as we used cross-sectional data here, causality cannot be securely assumed. At best, we were able to eliminate spurious candidates for causal relations. Causality assessment would require longitudinal data, collected repeatedly. Further studies may wish examine such concerns, using longitudinal network analysis. Thirdly, as we conducted the network analysis using a normative-community sample, the findings cannot be directly generalized to other samples, like specific racial and clinical groups. Fourthly, as we used web-based self-report measures, the findings may be confounded with common method variance, and may not be applicable to data collected via clinical interviews, or from other sources. Fifthly, as our findings are based on group-level analyses, it may not be directly applicable at the individual level. It is possible that some of the associations found in the current study may not be applicable to some individuals. Sixthly, as, the sample comprised mainly online employed male gamers, with non-pathological levels of gaming engagement, it is conceivable that the motivations for game playing in the study population may not actually represent motivations for IGD. This motivation assumptions applicability would be more certain if the sample had been pathological gamers. In this respect, other factors, such as psychiatric comorbidities, neurodevelopmental factors, sociocultural factors, and game-related factors, may exert a significant influence on the clinical picture of IGD (Ji et al., 2021 [63]). Seventhly, as our findings are based on a single study, there is need for more studies and replications before our findings can be generalized with confidence. Clearly, we need more network studies of the IGD symptoms, using longitudinal data, collected using multiple sources and methods and different racial and clinical groups. Individualized networks would also be beneficial for a comprehensive understanding of the IGD network. Despite these limitations, our findings do offer new insights on the structure of IGD symptoms, their relative importance, and their associations with different types of motivation that can be used effectively for theorizing, assessing and treating IGD.

## Supplementary Information

**Additional file 1.**

**Additional file 2.**

**Additional file 3.**

**Additional file 4.**

## Data Availability

Data is deposited as a supplementary file with the current document.

## Abbreviations

CFA: Confirmatory factor analysis
IRT: Item response theory
LCA: Latent class analysis
FMM: Factor mixture modeling
SDT: Self determination theory
OIT: Organismic integration subtheory
IGDS9-SF: Internet gaming disorder scale 9th edition - short form
SIMS: Situational intrinsic motivation scale
JASP: Jeffreys’ amazing statistics program
EBIC: Extended Bayesian information criterion
