# Supplementary material for: Network analyses of internet gaming disorder symptoms and their links with different types of motivation
Source: BMC Psychiatry. 2022 Jan 31;22:76. doi: 10.1186/s12888-022-03708-6 (PMC8802468; doi:10.1186/s12888-022-03708-6)
Supplement: Supplementary file 1 — Additional file 1. [file 12888_2022_3708_MOESM1_ESM.docx]

**Supplementary Table S1**

*Frequencies and Descriptive Statistics of Background Variables Collected in the Study*

| Variables | Frequencies/descriptive statistics |
| --- | --- |
| Employed | Frequency (percentage) = 532 (55.0%) |
| *Highest educational level* |  |
| Primary | Frequency (percentage) = 12 (1.2%) |
| Secondary | Frequency (percentage) = 251 (25.9%) |
| Technical | Frequency (percentage) = 85 (8.8%) |
| University | Frequency (percentage) = 603 (62.29%) |
| Others | Frequency (percentage) = 17 (176.8%) |
| Is involved in romantic relationships | Frequency (percentage) = 451 (46.6%) |
| Video game usage | Frequency (percentage) = 923 (95.4%) |
| Years playing preferred game | Mean (*SD*) = 18.12 (9.179); Ma/Min = 0/43 |
| Hours/week on preferred games | Mean (*SD*) = 9.48 (11.955); Ma/Min = 0/102 |

*Note*. *SD* = Standard Deviation; Max/Min = Maximum/Minimum.
