# Supplementary material for: Network analyses of internet gaming disorder symptoms and their links with different types of motivation
Source: BMC Psychiatry. 2022 Jan 31;22:76. doi: 10.1186/s12888-022-03708-6 (PMC8802468; doi:10.1186/s12888-022-03708-6)
Supplement: Supplementary file 2 — Additional file 2. [file 12888_2022_3708_MOESM2_ESM.docx]

**Supplementary Table S2**

*Mean and Standard Deviation Scores for all Network Variables*

|  | Mean | SD |
| --- | --- | --- |
| IGDS9-SF Symptoms | | |
| 1. Preoccupation (with gaming) | 2.77 | 1.239 |
| 2. Withdrawal symptoms (when gaming removed) | 1.89 | 1.055 |
| 3. Tolerance (need to spend more time in games) | 2.11 | 1.145 |
| 4. Loss of control (in participation in games) | 1.78 | 1.041 |
| 5. Giving up other activities (as a result of games) | 2.10 | 1.158 |
| 6. Continuation (despite knowledge of problems) | 1.68 | 1.077 |
| 7 Deception (people regarding the amount of gaming) | 1.40 | .875 |
| 8. Escape (using games to escape negative moods) | 3.05 | 1.345 |
| 9. Negative consequences (giving up relationship/opportunities because of games) | 1.38 | .840 |
| IGDS9-SF | | |
| Gaming Total | 18.15 | 7.114 |
| Situational Motivation Scale (SIMS) dimensions | | |
| Intrinsic Motivation | 21.63 | 4.764 |
| Identified Regulation | 18.59 | 4.864 |
| External Regulation | 9.09 | 4.965 |
| Amotivation | 9.09 | 4.903 |
