# Supplementary material for: Network analyses of internet gaming disorder symptoms and their links with different types of motivation
Source: BMC Psychiatry. 2022 Jan 31;22:76. doi: 10.1186/s12888-022-03708-6 (PMC8802468; doi:10.1186/s12888-022-03708-6)
Supplement: Supplementary file 3 — Additional file 3. [file 12888_2022_3708_MOESM3_ESM.docx]

**Supplementary Table S3**

*Weights Matrix between the IGDS9-SF Variables from the Network Analysis*

|  | PC | WS | T | LC | OA | C | D | E | NC |
| --- | --- | --- | --- | --- | --- | --- | --- | --- | --- |
| 1. Preoccupation (PC) | 0.000 |  |  |  |  |  |  |  |  |
| 2. Withdrawal symptoms (WS) | 0.250 | 0.000 |  |  |  |  |  |  |  |
| 3. Tolerance (T) | 0.273 | 0.203 | 0.000 |  |  |  |  |  |  |
| 4. Loss of control LC) | 0.005 | 0.370 | 0.147 | 0.000 |  |  |  |  |  |
| 5. Giving up other activities (OA) | 0.158 | 0.000 | 0.125 | 0.167 | 0.000 |  |  |  |  |
| 6. Continuation (C) | 0.000 | 0.000 | 0.079 | 0.181 | 0.172 | 0.000 |  |  |  |
| 7. Deception (D) | -0.041 | 0.062 | 0.000 | 0.118 | 0.086 | 0.389 | 0.000 |  |  |
| 8. Escape (E) | 0.109 | 0.086 | 0.184 | 0.103 | 0.068 | 0.022 | -0.061 | 0.000 |  |
| 9. Negative consequences (NC) | 0.073 | 0.044 | 0.000 | 0.063 | 0.000 | 0.287 | 0.323 | 0.000 | 0.000 |
