# Supplementary material for: Network analyses of internet gaming disorder symptoms and their links with different types of motivation
Source: BMC Psychiatry. 2022 Jan 31;22:76. doi: 10.1186/s12888-022-03708-6 (PMC8802468; doi:10.1186/s12888-022-03708-6)
Supplement: Supplementary file 4 — Additional file 4. [file 12888_2022_3708_MOESM4_ESM.docx]

**Supplementary Table S4**

*Weights between the IGDS9-SF Variables and the Situational Motivation Scale (SIMS) dimensions in the Network Analysis Involving both IGDS9-SF and SIMS Variables*

|  | Intrinsic Motivation | Identified Regulation | External Regulation | Amotivation |
| --- | --- | --- | --- | --- |
| Preoccupation | 0.109 | 0.000 | -0.047 | 0.000 |
| Withdrawal symptoms | 0.000 | 0.000 | 0.006 | 0.037 |
| Tolerance | 0.000 | 0.000 | 0.049 | 0.000 |
| Loss of control | 0.000 | 0.049 | 0.000 | 0.014 |
| Giving up other activities | 0.000 | -0.009 | 0.000 | 0.027 |
| Continuation | 0.000 | 0.000 | 0.050 | 0.045 |
| Deception | -0.058 | 0.000 | 0.039 | 0.012 |
| Escape | 0.095 | 0.000 | -0.071 | 0.000 |
| Negative consequences | -0.012 | -0.093 | 0.016 | 0.149 |
